# Large Bowel Endometriosis Mimicking Colorectal Cancer

**DOI:** 10.1155/2020/7014107

**Published:** 2020-07-29

**Authors:** Laís Marques Mota, Bruno Salomão Hirsch, Renato Seligman

**Affiliations:** ^1^Graduated in Internal Medicine by Hospital de Clínicas de Porto Alegre, Rua Ramiro Barcelos, 2350 Santa Cecília, Porto Alegre 96020-700, RS, Brazil; ^2^Graduated in Gastroenterology by Hospital de Clínicas de Porto Alegre, Rua Ramiro Barcelos, 2350 Santa Cecília, Porto Alegre 96020-700, RS, Brazil; ^3^Universidade Federal do Rio Grande do Sul, Rua Ramiro Barcelos, 2400-Santa Cecilia, Porto Alegre 90035-007, RS, Brazil; ^4^Hospital de Clínicas de Porto Alegre, Rua Ramiro Barcelos, 2350-Santa Cecilia, Porto Alegre 90035-007, RS, Brazil

## Abstract

Endometriosis is characterized by the presence of endometrial tissue outside the uterus, with 1–7% prevalence in the female population. It is observed in various locations of the human body, and large bowel endometriosis is the most common extrapelvic site, affecting about 5 to 12% of all women who present endometriosis. This study aimed to report an interesting images related to stenosing large bowel endometriosis that was possible to be diagnosed only by surgical intervention. Hence, this pathology is a diagnostic challenge and should be remembered between differential diagnoses of nonspecific or even alarming symptoms of the gastrointestinal tract.

## 1. Introduction

Endometriosis is characterized by the presence of endometrial tissue outside the uterus, with 1–7% prevalence in the female population. It is observed in various locations of the human body, and large bowel endometriosis is the most common extrapelvic site, affecting about 5 to 12% of all women who present endometriosis [1, 2].

The clinical manifestations include a broad spectrum of symptoms, from the most classic ones such as dyspareunia, infertility, and dysmenorrhea to the less frequent symptoms such as urinary or intestinal complaints. Thus, nonspecific signs or symptoms of the gastrointestinal tract-abdominal pain, rectal bleeding, palpable abdominal mass, diarrhea, or constipation may represent difficulties in the differential diagnosis of a benign disease that eventually has malignant behavior [3, 4]. Except in cases of rectal nodules, intestinal endometriosis cannot be diagnosed only by physical examination [5].

## 2. Case Description

A 51-year-old female, from Guaíba, South of Brazil, was referred to a gastroenterology outpatient clinic at the Hospital de Clínicas de Porto Alegre. She had noticed a decrease in her bowel habits (evacuations every three days), as well as sharp and long stools, with progressive worsening during one year period. She also had intermittent episodes of elimination of red blood mixed with feces, every 3–4 weeks; she denied associated abdominal pain, weight loss, or night sweats. On physical examination, blood pressure was 124 × 78 mmHg, heart rate 80bpm, and respiratory rate 14 and normal pulmonary and cardiac auscultations were performed. The abdomen was painless, without palpable masses or visceromegaly, and normal bowel sounds were present. The rectal examination showed no nodules, bleeding, or masses, and there was no palpable enlargement of the lymph nodes.

Personal and family history: symptomatic cholelithiasis (awaiting surgery), two previous cesarean sections (G2P2), and hysterectomy in 2011 (fibroids). She denied continued use of medications, alcoholism, smoking, or drug use. She had no family history of gastrointestinal tract disease or any type of cancer.

**Figure 1:**
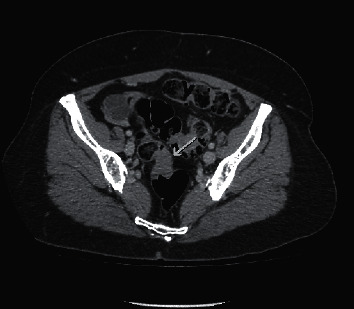
Abdomen tomography with stenosing lesions in the upper rectum and sigmoid presenting impregnation with contrast, as shown by the pointing arrow.

**Figure 2:**
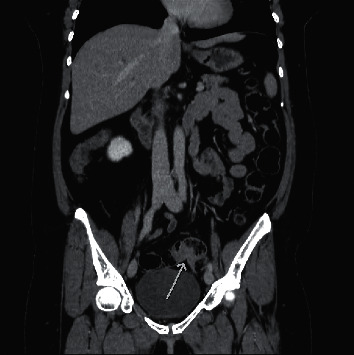
Coronal image of abdomen tomography with stenosing lesions in sigmoid presenting impregnation with contrast, as shown by the pointing arrow.

**Figure 3:**
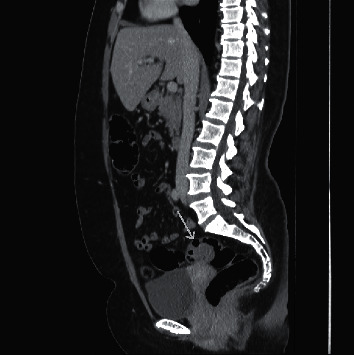
Sagital image of abdomen tomography with stenosing lesions in the upper rectum presenting impregnation with contrast, as shown by the pointing arrow.

Investigations were performed initially with laboratory tests (blood count, renal function, electrolytes, liver function tests were normal). Afterwards, she underwent tomography of the abdomen (CT), showing two areas of irregular parietal thickening, one in the upper rectum with approximately 3.0 cm extension and the other in the middle segment of the sigmoid colon, measuring about 2.5 cm in length, presenting impregnation with contrast (Figures 1–3). These lesions were stenosing but did not determine significant upstream intestinal obstruction. Among the diagnostic possibilities, a synchronic colorectal neoplasia was highlighted. An opaque enema was performed before performing a colonoscopy that confirmed the findings of the CT scan. During rectosigmoidoscopy, the device was inserted up to 30 cm from the anal margin, where a concentric stenosis was observed, covered by macroscopically normal mucosa, preventing the progression of the device. At 15 cm from the anal margin, a caliber reduction area covered by irregular and friable mucosa was observed. A biopsy specimen was performed and the anatomopathological study resulted in colonic mucosa without relevant alterations.

The surgical team evaluated the patient and decided to perform a rectosigmoidectomy with cholecystectomy, which had no complications and evidenced the two stenoses mentioned in the tomography with surrounding fibrotic adhesions. The anatomopathological study of the surgical specimen evidenced intestinal wall endometriosis, compromising submucosa and internal and external muscular layers, with fibrosis. The patient subsequently evolved with complete improvement of the referred symptoms.

## 3. Discussion

Low intestinal bleeding, represented in the case reported as hematochezia, is a sign of different pathologies, from benign to malignant. What is noticeable in this case are the intestinal cycling changes that the patient presented, rectal bleeding every 3–4 weeks, which should alert to the diagnosis of endometriosis. Inflammatory bowel disease, irritable bowel syndrome, neoplastic diseases, diverticular disease, and hemorrhoidal disease are part of the differential diagnosis [6].

Some patients have multiple lesions of endometriosis involving more than one intestinal segment [5]. In this case, two areas of irregular parietal thickening were found, one in the upper rectum and the other in the middle segment of the sigmoid colon, which were initially interpreted as suspect of synchronic neoplasia but which, in fact, were found to be compatible with endometriosis [5].

It is known that the extension of the disease is not necessarily related to the intensity of the symptoms; many women are asymptomatic with extensive disease by incidental finding, as some women with mild illness have disabling symptoms [4]. In addition, deep infiltrative endometriosis (DIE) of the intestine invades at least at the level of the serosa and the muscular layer itself [7]. The patient in the case had no symptoms until a year ago, including most of her reproductive life without the classics symptoms of endometriosis (dyspareunia, dysmenorrhea, and infertility) and the anatomopathological study indicated as intestinal wall DIE, compromising submucosa and internal and external muscular layers, with fibrosis, and therefore, evidenced extensive disease by the pathology classification.

In addition, endometrial tissue outside the uterus is probably an underdiagnosed disease according to some reports, presumably some patients with functional disorders such as irritable bowel syndrome may actually have intestinal endometriosis, since the symptoms of both pathologies are nonspecific.Thus, the diagnosis of endometriosis is relevant because it offers a simple treatment in menacme, for example, using hormone therapy.

## 4. Conclusion

Large bowel endometriosis is a diagnostic challenge and should be remembered between differential diagnoses of nonspecific or even alarming symptoms of the gastrointestinal tract.
